# Quantitative Structure-Activity Relationship Study of Antioxidant Tripeptides Based on Model Population Analysis

**DOI:** 10.3390/ijms20040995

**Published:** 2019-02-25

**Authors:** Baichuan Deng, Hongrong Long, Tianyue Tang, Xiaojun Ni, Jialuo Chen, Guangming Yang, Fan Zhang, Ruihua Cao, Dongsheng Cao, Maomao Zeng, Lunzhao Yi

**Affiliations:** 1 Guangdong Provincial Key Laboratory of Animal Nutrition Control, National Engineering Research Center for Breeding Swine Industry, Subtropical Institute of Animal Nutrition and Feed, College of Animal Science, South China Agricultural University, Guangzhou 510642, China; hongronglong@stu.scau.edu.cn (H.L.); tianyue.tang@wur.nl (T.T.); nnnxjun@stu.scau.edu.cn (X.N.); chenjialuo@stu.scau.edu.cn (J.C.); 18319772208@163.com (G.Y.); Fanzhang@stu.scau.edu.cn (F.Z.); caoruihua@stu.scau.edu.cn (R.C.); 2 Xiangya School of Pharmaceutical Sciences, Central South University, Changsha 410083, China; oriental-cds@163.com; 3 State Key Laboratory of Food Science and Technology, International Joint Laboratory on Food Safety, Jiangnan University, Wuxi 214122, China; mmzeng@jiangnan.edu.cn; 4 Yunnan Food Safety Research Institute, Kunming University of Science and Technology, Kunming 650500, China; yilunzhao@kmust.edu.cn

**Keywords:** quantitative structure-activity relationship, QSAR, antioxidant tripeptides, model population analysis, amino acid descriptors

## Abstract

Due to their beneficial effects on human health, antioxidant peptides have attracted much attention from researchers. However, the structure-activity relationships of antioxidant peptides have not been fully understood. In this paper, quantitative structure-activity relationships (QSAR) models were built on two datasets, i.e., the ferric thiocyanate (FTC) dataset and ferric-reducing antioxidant power (FRAP) dataset, containing 214 and 172 unique antioxidant tripeptides, respectively. Sixteen amino acid descriptors were used and model population analysis (MPA) was then applied to improve the QSAR models for better prediction performance. The results showed that, by applying MPA, the cross-validated coefficient of determination (Q^2^) was increased from 0.6170 to 0.7471 for the FTC dataset and from 0.4878 to 0.6088 for the FRAP dataset, respectively. These findings indicate that the integration of different amino acid descriptors provide additional information for model building and MPA can efficiently extract the information for better prediction performance.

## 1. Introduction

Bioactive peptides, usually containing 2–20 amino acid residues, are typically derived from the enzymatic hydrolysis of proteins [1]. They are inactive within the sequence of proteins, but they can exert various physiological functions after release. Antioxidant peptides are one of the most important groups of bioactive peptides, which can prevent oxidative stress and they have notable contributions to human health [2]. Antioxidant peptides have been isolated and purified from sources, such as cereals, milk, meat, and fish [3]. The methods to assess the antioxidant capacities of peptides include the Trolox equivalent antioxidant capacity (TEAC), the ferric ion reducing antioxidant power (FRAP), the 2,2-diphenyl-1-picrylhydrazyl radical-scavenging capacity (DPPH), the oxygen radical absorbance capacity (ORAC), the total radical trapping antioxidant parameter (TRAP), etc. [4]. However, it is impossible to test all of the peptides to find valid antioxidants, when considering the large number of theoretical possible peptides, i.e., 400 dipeptides, 8000 tripeptides, 160,000 tetrapeptides, etc.

The activities of peptides are determined by the amino acid compositions, sequences, and structures. Quantitative structure-activity relationship (QSAR), which is a well-recognized tool for estimating chemical activities, has been widely applied for bioactive peptides prediction [5]. The QSAR models have been successfully built on ACE-inhibitory peptides [6], antimicrobial peptides [7], antioxidant peptides [8,9,10], antitumor peptides [11], bitter peptides [12], and etc. The QSAR study of antioxidant peptides mainly focused on di and tripeptides, because they can be absorbed intact from the intestinal lumen into the bloodstream and then produce biological effects at the tissue level [13]. When compared to dipeptides, tripeptides were reported to exhibit higher levels of antioxidant activity [14]. Besides, tripeptides had much larger structural diversity than dipeptides, which is a good property for developing multifunctional food additives [15].

The prediction performances need to be further improved, although plenty of QSAR models have been built on antioxidant peptides. The relationship between peptide structure and antioxidant activity is still unclear. This may be due to the restriction of model building methods. Model population analysis (MPA) provides a new strategy of model building, which is to use multi-models instead of a single model to improve prediction ability and interpretability [16,17]. Previous studies showed that, through the application of MPA strategy, the performance of regression models could be improved [6,18].

In this study, we built QSAR models based on two antioxidant tripeptides datasets. The first dataset contains 214 artificially designed tripeptides and the second dataset contains 172 β-Lactoglobulin derived tripeptides, which represent designed or food originated tripeptides, respectively. 16 amino acid descriptors were used to construct sophisticated data for the comprehensive information of peptides. The MPA strategy was applied to extract useful information from the data and to optimize the models. The aim of this study is not to build a new set of descriptors, but to integrate different descriptors under the framework of MPA for better QSAR model performance on antioxidant tripeptides data. The improved method for QSAR modelling will help in discovering new antioxidant tripeptides for future drugs or food additives.

## 2. Results

### 2.1. FTC Dataset

The results of QSAR models on the FTC dataset are displayed in Table 1. Before outlier elimination, the largest Q^2^ value of 0.4901 is obtained on the VSW descriptor. After outlier elimination, the HSEHPCSV descriptor showed the largest Q^2^ value of 0.6170 among the 16 amino acid descriptors. The integration of 16 descriptors gave rise to an improvement of the model performance (Q^2^ = 0.6818). Finally, the model prediction performance was further improved (Q^2^ = 0.7471) after variable selection while using the BOSS method.

**Figure 1:**
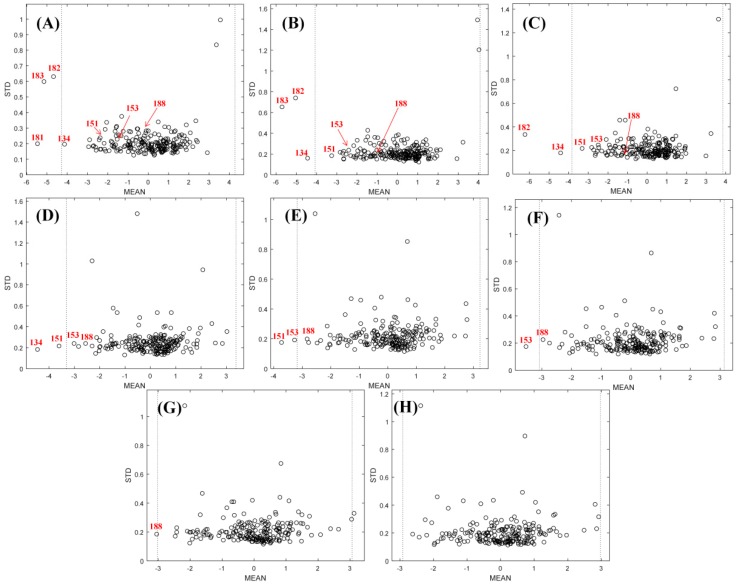
The process of model population analysis (MPA)-based outlier elimination on the FTC dataset of integrated descriptors. The dashed line is defined as the boundary for outliers, which is mean ± 3× standard deviation of prediction errors. (**A**) No outlier was eliminated, (**B**) sample No. 181 was eliminated, (**C**) sample No. 183 was eliminated, (**D**) sample No. 182 was eliminated, (**E**) sample No. 134 was eliminated, (**F**) sample No. 151 was eliminated, (**G**) sample No. 153 was eliminated, and (**H**) sample No. 188 was eliminated and all of the outliers were removed.

In this study, an MPA-based outlier elimination procedure [19] was carried out to remove outliers one by one (Figure 1). For the integrated data, samples of no. 181, 183, 182, 134, 151, 153, and 188 were removed in sequence. Finally, all of the samples were within the range according to the three-sigma rule after outlier removal (Figure 1H, dashed line).

**Figure 2:**
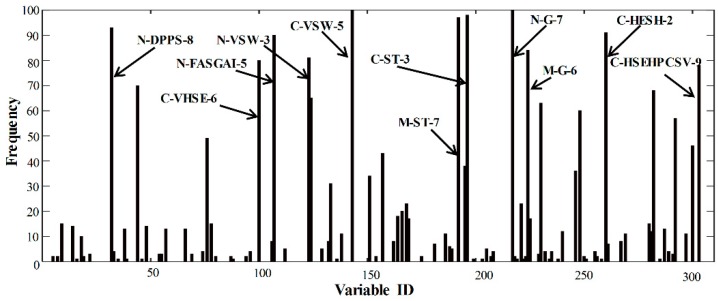
Frequency of variables selected by the bootstrapping soft shrinkage (BOSS) method on the FTC dataset in 100 runs. The higher frequency denotes higher variable importance. The top 11 variables with frequency larger than 75 were marked in the figure.

Figure 2 showed the selected variables by the BOSS method in 100 runs. The variables being selected more frequently reflect high variable importance. The top 11 variables (frequency>75), in descending order, were as follows: C-VSW-5 = N-G-7 > C-ST-3 > M-ST-7 > N-DPPS-8 > C-HESH-2 > N-FASGAI-5 > M-G-6 > N-VSW-3 > C-VHSE-6 >C-HSEHPCSV-9, which are marked on Figure 2. All the top 11 variables originated from the best preformed amino acid descriptors, i.e., HSEHPCSV, ST-scale, HESH, G-scale, FASGAI, and DPPS (Table 1). It showed that the ultimate model has the merit of the best performed models that were constructed by single amino acid descriptors.

### 2.2. FRAP Dataset

The results of QSAR models on FRAP dataset are displayed in Table 2. Before logarithmic transformation of response vector Y, the largest Q^2^ value of 0.1408 is obtained on 5Z-scale descriptor. The low Q^2^ value indicated that the tripeptide structures and their antioxidant activities that were evaluated by FRAP assay did not share a linear relationship. After logarithmic transformation, the VHSE descriptor showed the largest Q^2^ value of 0.4878. Through integrating the 16 descriptors, the Q^2^ value was increased slightly to 0.4953. The prediction performance of the model was promoted after variable selection using the BOSS method (Q^2^ = 0.6088). It indicated that a linear relationship between the structures and the activities was built after the logarithmic transformation of Y and the MPA strategy was efficient in improving the model.

**Figure 3:**
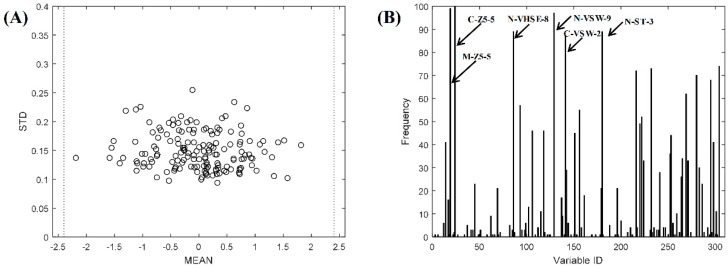
The result of QSAR model building on the FRAP dataset. (**A**) The result of MPA-based outlier detection on the FRAP dataset of integrated descriptors. No outlier was detected. (**B**) Frequency of variables selected by the BOSS method on the FRAP dataset in 100 runs. The higher frequency denotes higher variable importance. The six top variables with frequency larger than 75 are marked in the figure.

Similarly, an MPA-based outlier elimination procedure was carried out on the FRAP dataset. No outlying sample was detected, since all of the samples gather within the range according to the three-sigma rule (Figure 3A, dashed line). The important variables that were selected by BOSS are displayed in Figure 3B. The six most important variables (frequency > 75) are C-Z5-5, M-Z5-5, N-VSW-9, N-VHSE-8, N-ST-3, and C-VSW-2, respectively. Most of the important variables originated from three well performed descriptors, i.e., VHSE, 5Z-scale, and ST-scale. However, there still some variables selected from the poorly performed descriptor, such as VSW. It suggested that descriptors with poor performance also contained useful information for model building.

## 3. Discussion

### 3.1. Comparison with the Reported Models

For the FTC dataset, our method showed higher prediction accuracy (Q^2^ = 0.7471), when compared to the previous report (Q^2^ = 0.6310) [20]. Note that 41 sample were eliminated as outliers in the previous study, while only seven outliers were eliminated in this study. A much larger number of samples was used in our model, which is more representative. It showed that our method exhibited a model with higher prediction performance and the relatively larger applicability domain.

Similarly, for FRAP dataset, our method showed a higher prediction accuracy (Q^2^ = 0.6008) when compared to the previous report (Q^2^ = 0.5410) [21]. It should be noted that, in the previous study, five samples with the highest activities and 14 inactive samples were removed, while in our study, only inactive samples were removed. Thus, our model showed improved prediction accuracy and enlarged applicability domain.

### 3.2. Relationship between Antioxidant Activities and Peptide Structures

Previous studies showed that the N-terminus and C-terminus amino acids are important in relating to antioxidant activities [20]. Our results are in agreement with the previous findings that most of the important variables that were selected by BOSS originated from the N-terminus or C-terminus (Figure 2 and Figure 3B). In addition, studies showed that tripeptides containing Cys (C), Trp (W), and Tyr (Y) residues exhibited strong antioxidant activities [8,10]. Tripeptides YHY and LTC, for the two datasets, respectively, having the highest antioxidant activities is confirmed by our study.

On the FTC dataset, a linear relationship between antioxidant activities and peptide structures was constructed. However, on the FRAP dataset, the relationship was only built on the log-transformed activities and structure properties. It indicates that the antioxidant activity and peptide structures on the FRAP dataset exhibits a non-linear relationship. Data transformation is crucial before model building on this kind of data. The different performance of the two datasets may be attributed to the structure diversities of peptides. In the FTC dataset, tripeptides contain either the His or Tyr residue, which have similar structures, while the structure diversity in the FRAP dataset is much larger.

### 3.3. The Integration of Amino Acid Descriptors

A number of amino acid descriptors have been developed and applied in the QSAR studies of bioactive peptides. Each descriptor has its merits and demerits. Our study shows that an optimal descriptor does not exist. Instead, all of the descriptors are data dependent, which means that each descriptor performs well on different datasets. It makes the researches difficult to select descriptors. By integrating different descriptors, each one can contribute particular information to the model and create a new possibility for further improvement of the model. Subsequently, the next question has become how to efficiently extract information from different descriptors and to get rid of the redundancy of the data? Model population analysis (MPA) may provide a solution for that. It uses multi-models instead of a single model for prediction. Each sub-model contains a random combination of different descriptors. Through statistical analysis of the sub-model outcomes, the informative variables from the descriptors are extracted and an optimized descriptor combination is obtained [22]. Finally, the optimized model performs better than any of the single descriptor model, as it is shown in Table 1 and Table 2. To summarize, the aim of this study is not to build a new set of descriptors, but to provide a general framework to integrate different descriptors. The framework can take in any newly developed descriptor and fit on different datasets. The more diverse the integrated descriptors are, the better performance the model can be.

## 4. Materials and Methods

### 4.1. Data Collection

#### 4.1.1. Ferric Thiocyanate (FTC) Dataset

A dataset of 214 antioxidant tripeptides that contain either His or Tyr residue was obtained from the published literatures [20,23]. All of the tripeptides were chemically synthesized using solid phase Fmoc Chemistry and their antioxidant activities were measured by the FTC method [23]. Test samples (500 μg) in 0.5 mL of deionized water were mixed with linoleic acid emulsion (1.0 mL, 50 mM) and phosphate buffer (1.0 mL, 0.1 M) in glass test tubes (5 mL). The tubes were sealed with silicon rubber caps and then kept at 60 °C in the dark. 50 μL reaction mixtures were taken out at different intervals during incubation. The degree of oxidation was measured by sequentially adding ethanol (2.35 mL, 75%), ammonium thiocyanate (50 μL, 30%), and ferrous chloride (50 μL, 20 mM in 3.5% HCl). After the mixture had stood for 3 min, the absorbance of the solution was measured at 500 nm with a Jasco model Ubest 30 spectrophotometer (Tokyo, Japan). A control was performed containing the same contents with test sample but without the peptides. The number of days that was taken to attain the absorbance of 0.3 was defined as the induction period. The relative activities were calculated by dividing the induction period of test samples by that of the control (Table 3). All of the experiments were carried out in triplicate and averaged.

#### 4.1.2. Ferric-reducing Antioxidant Power (FRAP) Dataset

A dataset of 172 antioxidant tripeptides were derived from β-Lactoglobulin, where all possible tripeptides were collected based on its amino sequence [21]. All of the tripeptides were chemically synthesized while using solid phase Fmoc Chemistry and their antioxidant activities were evaluated using the FRAP assay [24]. Ten microliters of 100 mmol/mL tripeptide solution were incubated at 37 °C with 100 μL of FRAP reagent, containing 10 mmol/L of 2,4,6-tripyridyl-s-triazine and 20 mmol/L of FeCl_3_. The absorption values were read at a wavelength of 570 nm using a microplate reader (Model 680, Bio-Rad, Hercules, CA, USA) after 10 min reaction. Aqueous Fe^2+^ solutions at concentrations that ranged from 10 to 1000 μmol/L were used to produce a calibration curve. The results were expressed as micromoles Fe^2+^ equivalents per mole of the sample based on the standard curve. All of the experiments were carried out in triplicate and then averaged. The activities were logarithmic transformed prior to modeling, where 14 inactive peptides (activity = 0) were removed (Table 4). The measured activities before logarithmic transformation were displayed in Table S1.

The two datasets are representative for artificially designed or food protein originated tripeptides, respectively. Both of the datasets have been used for building QSAR models before. Thus, it is suitable for model comparison.

### 4.2. Data Processing

The tripeptide sequences were transformed into X-matrices using 16 amino acid descriptors, respectively, while the dependent variable Y-vectors represents the relative activities of peptides. These descriptors include Z-scale, 5Z-scale, DPPS, MS-WHIM, ISA-ECI, VHSE, FASGAI, VSW, T-scale, ST-scale, E-scale, V-scale, G-scale, HESH, and HSEHPCSV, as is shown in Table 5. They are the most frequently used amino acid descriptors in the QSAR study of bioactive peptides. The peptide structure is characterized by describing amino acids within the sequence. For example, Z-scale descriptor, containing three parameters (Z1, Z2, and Z3), would generate nine variables (3 parameters × 3 amino acids) for tripeptides. To clearly label each variable, we used a unified rule to name them. The amino acid at the N-terminus was designated as N, the C-terminus amino acid was designated as C, and the middle amino acid was designated as M. Thus, the nine variables that were generated by Z-scale descriptor were labeled as N-Z-1, N-Z-2, N-Z-3, M-Z-1, M-Z-2, M-Z-3, C-Z-1, C-Z-2, and C-Z-3, respectively. The 16 descriptors were integrated to build an X-matrix, which contained 306 variables (V1-V306), with the correspondence, as follows: Z-scale (V1-V9), 5Z-scale (V10-V24), DPPS (V25-V54), MS-WHIM1 (V55-V63), MS-WHIM2 (V64-V72), ISA-ECI (V73-V78), VHSE (V79-V102), FASGAI (V103-V120), VSW (V121-V147), E-scale (V148-V162), T-scale (V163-V177), ST-scale (V178-V201), V-scale (V202-V210), G-scale (V211-V234), HESH (V235-V270), and HSEHPCSV (V271-V306), respectively.

### 4.3. QSAR Model Building

Partial least squares (PLS) regression [40] was used to build the connection between the peptide structure descriptions (variables, X-matrices) and the relative activities (responses, Y-vectors). It was implemented using MATLAB software (Version R2015a, the MathWorks, Inc., Natick, MA, USA). All of the variables were auto-scaled to unit variance and all of the responses were mean-centered prior to model building. The models were validated using cross-validation and the optimal number of PLS components were chosen based on a statistic, called Q^2^, which is the cross-validated R^2^, referring to the predictive ability of the model. R^2^ is the coefficient of determination, providing an estimate of the model fit.

MPA was applied to optimize the model through outlier elimination and variable selection. It is a framework for model building that utilizes multiple models instead of a single model to construct results [16,17]. Generally, it worked, as follows: (1) firstly, a random resampling procedure was applied to obtain sub-datasets; (2) then, sub-models were built based on the sub-datasets; and, (3) finally, a statistical analysis was used to extract useful information from the outcome of sub-models. In the present study, MPA was utilized for outlier detection and variable selection.

The MPA-based outlier detection method [19] was applied to remove the outlying samples from measured data. To begin with, 1000 sub-datasets were generated through random reselecting of 80% samples in sample space. Subsequently, for each sub-dataset, a PLS regression model was built and the prediction error for each sample was recorded. The mean of prediction errors was used as the basis for outlier detection and a three-sigma rule was applied to define the boundary, as it is reported previously [6]. The bootstrapping soft shrinkage (BOSS) method [18] was applied to select informative variables from the pool of descriptors. It is also based on the idea of MPA. Firstly, 1000 sub-datasets were obtained using bootstrap resampling in the variable space. Afterwards, 1000 PLS models were built based on the sub-datasets and the regression coefficients were extracted. In the next step, weighted bootstrap resampling was used to regenerate sub-datasets and to rebuild sub-model. The resampling procedure was repeated until all of the uninformative variables were eliminated.

## 5. Conclusions

In this study, we have constructed QSAR models on two datasets of antioxidant tripeptides, i.e., FTC dataset and FRAP dataset. After the integration of 16 amino acid descriptors and utilization of the MPA strategy for model building, the Q^2^ values were enlarged from 0.6170 to 0.7471 and from 0.4878 to 0.6088, respectively. The results show that the MPA framework is powerful in QSAR model building on antioxidant tripeptides data. The framework can also be applied to investigate the structure and activity relationships of other types of bioactive peptides and to integrate more different molecular descriptors.

## Figures and Tables

**Table 1:** Comparisons among different quantitative structure-activity relationships (QSAR) models on ferric thiocyanate (FTC) dataset ^a^.

Descriptors	Before Outlier Elimination			After Outlier Elimination			
	Q^2^	R^2^	optPC	Q^2^	R^2^	optPC	Outlier
HSEHPCSV	0.3861	0.5781	4	**0.6170**	0.7338	20	183, 182, 181, 134
ST-scale	0.4268	0.5733	12	0.5993	0.6844	13	183, 182, 181, 134
HESH	0.4091	0.5366	2	0.5968	0.7047	10	183, 181, 182, 134, 129
VSW	**0.4901**	0.5771	3	0.5925	0.6768	5	181, 183, 182, 134, 151
G-scale	0.4516	0.5527	6	0.5843	0.6574	9	181, 183, 182, 134, 118
FASGAI	0.4814	0.5457	5	0.5544	0.6130	6	129, 181, 128
DPPS	0.4740	0.5637	7	0.5379	0.6278	8	181, 182, 183, 134
E-scale	0.4956	0.5451	4	0.5144	0.5582	4	181, 182, 183, 112
5Z-scale	0.3903	0.4626	12	0.3974	0.4653	9	181, 182, 183, 172
VHSE	0.4265	0.5432	12	0.3974	0.514	8	181, 182, 183, 172
T-scale	0.3280	0.4215	9	0.3728	0.4362	9	181, 182, 183
V-scale	0.3371	0.3785	5	0.3070	0.3458	6	181, 183, 182
Z-scale	0.2814	0.3398	4	0.2678	0.3415	4	181
ISA-ECI	0.1493	0.1916	6	0.1572	0.1836	6	183, 182, 181
MS-WHTM1	0.0736	0.1488	3	0.1036	0.1678	3	181, 183, 182
MS-WHTM2	0.0775	0.1445	3	0.0882	0.1617	3	181, 182, 183
Integrated descriptors	0.4811	0.5843	3	0.6818	0.7964	8	181, 183, 182, 134, 151, 153, 188
BOSS				**0.7471** ± 0.0032	0.7931 ± 0.0062	9.72 ± 3.2199	

^a^ R^2^ is the coefficient of determination; Q^2^ is the cross-validated R^2^; optPC is optimal principal components for PLS regression model; the results of BOSS are shown in the form of mean value ± standard deviation in 100 runs; the top ranked Q^2^ scores were marked in bold.

**Table 2:** Comparisons among different QSAR models on FRAP dataset ^a^.

Descriptors	Before Logarithmic Transformation			After Logarithmic Transformation		
	Q^2^	R^2^	optPC	Q^2^	R^2^	optPC
VHSE	0.0042	0.2655	3	**0.4878**	0.6122	6
5Z-scale	**0.1408**	0.3177	2	0.4809	0.5568	3
DPPS	0.0059	0.2290	3	0.4147	0.5463	4
ST-scale	0.0263	0.3220	8	0.3968	0.5410	9
FASGAI	0.0470	0.2753	2	0.3735	0.5006	4
E-scale	0.0560	0.2521	1	0.3714	0.4734	5
HESH	0.0444	0.2818	10	0.3668	0.5290	3
HSEHPCSV	0.0259	0.2475	7	0.3624	0.4952	3
G-scale	0.1066	0.2334	5	0.2836	0.3850	1
VSW	0.0130	0.3071	1	0.2382	0.4361	2
MS-WHTM2	0.0342	0.0370	3	0.1728	0.2594	3
MS-WHTM1	0.0452	0.0329	9	0.1207	0.1941	4
T-scale	0.0682	0.0706	2	0.0750	0.2129	10
V-scale	0.0293	0.0748	4	0.0699	0.1495	1
Z-scale	0.0052	0.1445	1	0.0301	0.1456	6
ISA-ECI	0.0242	0.0141	1	0.0071	0.0411	1
Integrated descriptors	0.1069	0.4212	3	0.4953	0.6423	3
BOSS				**0.6088** ± 0.0041	0.6655 ± 0.0094	3.5100 ± 2.5086

^a^ R^2^ is the coefficient of determination; Q^2^ is the cross-validated R^2^; optPC is optimal principal components for PLS regression model; the results of BOSS are shown in the form of mean value ± standard deviation in 100 runs, the top ranked Q^2^ scores were marked in bold.

**Table 3:** Sequences and antioxidant activities of tripeptides on ferric thiocyanate (FTC) dataset ^a^.

No.	Sequence	Activity	No.	Sequence	Activity	No.	Sequence	Activity	No.	Sequence	Activity	No.	Sequence	Activity	No.	Sequence	Activity
1	LHA	3.918	37	PHA	5.793	73	RHA	5.205	109	DHH	0.9045	145	HHH	0.0635	181	YHY	9.886
2	LHD	3.593	38	PHD	4.622	74	RHD	3.304	110	EHH	0.9045	146	HHK	0.0635	182	YKY	9.886
3	LHE	6.136	39	PHE	6.152	75	RHE	5.096	111	HHH	0.0000	147	HHR	0.0635	183	YRY	9.886
4	LHF	3.628	40	PHF	3.916	76	RHF	3.300	112	KHH	0.0000	148	HHA	0.0680	184	YAY	3.607
5	LHG	6.697	41	PHG	5.197	77	RHG	5.725	113	AHH	2.020	149	HHI	0.0680	185	YIY	3.607
6	LHH	4.836	42	PHH	6.051	78	RHH	3.296	114	IHH	2.020	150	HHL	0.0680	186	YLY	3.607
7	LHI	6.531	43	PHI	4.916	79	RHI	4.806	115	FHH	1.803	151	HHF	3.612	187	YFY	2.233
8	LHK	4.225	44	PHK	3.426	80	RHK	2.694	116	WHH	1.803	152	HHW	3.612	188	YWY	2.233
9	LHL	5.920	45	PHL	5.311	81	RHL	3.501	117	YHH	1.803	153	HHY	3.612	189	YYY	2.233
10	LHM	4.504	46	PHM	3.714	82	RHM	3.218	118	GHH	1.089	154	HHG	0.3170	190	YGY	3.366
11	LHN	5.148	47	PHN	6.061	83	RHN	5.713	119	NHH	1.089	155	HHN	0.3170	191	YNY	3.366
12	LHQ	4.136	48	PHQ	3.718	84	RHQ	3.108	120	QHH	1.089	156	HHQ	0.3170	192	YQY	3.366
13	LHR	5.184	49	PHR	4.751	85	RHR	4.302	121	MHH	2.015	157	HHM	0.0817	193	YMY	1.780
14	LHS	4.293	50	PHS	4.042	86	RHS	3.386	122	SHH	1.320	158	HHS	0.0862	194	YSY	3.447
15	LHT	5.584	51	PHT	6.247	87	RHT	5.987	123	THH	1.320	159	HHT	0.0862	195	YTY	3.447
16	LHV	3.481	52	PHV	3.335	88	RHV	3.206	124	CHH	0.9369	160	HHC	0.1277	196	YCY	3.087
17	LHW	6.791	53	PHW	6.535	89	RHW	5.878	125	HDH	1.477	161	DYY	3.417	197	YYD	4.116
18	LHY	4.203	54	PHY	4.227	90	RHY	3.378	126	HEH	1.477	162	EYY	3.417	198	YYE	4.116
19	LWA	1.192	55	PWA	1.396	91	RWA	1.212	127	HHH	0.0441	163	HYY	2.257	199	YYH	5.303
20	LWD	1.717	56	PWD	1.096	92	RWD	0.9091	128	HKH	0.0441	164	KYY	2.257	200	YYK	5.303
21	LWE	1.717	57	PWE	1.096	93	RWE	1.091	129	HRH	0.0441	165	RYY	2.257	201	YYR	5.303
22	LWF	1.414	58	PWF	0.9192	94	RWF	0.9091	130	HAH	0.9518	166	AYY	3.071	202	YYA	3.344
23	LWG	1.313	59	PWG	2.687	95	RWG	1.717	131	HIH	0.9518	167	IYY	3.071	203	YYI	3.344
24	LWH	3.212	60	PWH	1.184	96	RWH	1.091	132	HLH	0.9518	168	LYY	3.071	204	YYL	3.344
25	LWI	1.111	61	PWI	1.396	97	RWI	1.232	133	HFH	2.026	169	FYY	1.911	205	YYF	4.050
26	LWK	1.899	62	PWK	0.4066	98	RWK	0.6061	134	HWH	2.026	170	WYY	1.911	206	YYW	4.050
27	LWL	0.6060	63	PWL	1.096	99	RWL	3.212	135	HYH	2.026	171	YYY	1.911	207	YYY	4.050
28	LWM	1.394	64	PWM	0.7955	100	RWM	0.7273	136	HGH	0.8318	172	GYY	5.071	208	YYG	2.996
29	LWN	1.313	65	PWN	2.104	101	RWN	2.404	137	HNH	0.8318	173	NYY	5.071	209	YYN	2.996
30	LWQ	2.505	66	PWQ	1.202	102	RWQ	0.6061	138	HQH	0.8318	174	QYY	5.071	210	YYQ	2.996
31	LWR	2.909	67	PWR	2.705	103	RWR	2.384	139	HMH	0.8734	175	MYY	1.991	211	YYM	2.103
32	LWS	2.020	68	PWS	1.096	104	RWS	0.8081	140	HSH	0.7304	176	SYY	3.070	212	YYS	3.983
33	LWT	2.020	69	PWT	2.598	105	RWT	3.818	141	HTH	0.7304	177	TYY	3.070	213	YYT	3.983
34	LWV	1.616	70	PWV	1.008	106	RWV	0.6061	142	HCH	0.9747	178	CYY	0.4699	214	YYC	0.6369
35	LWW	3.515	71	PWW	2.899	107	RWW	2.707	143	HHD	0.1877	179	YDY	3.047			
36	LWY	2.222	72	PWY	1.114	108	RWY	0.8081	144	HHE	0.1877	180	YEY	3.047	□	□	□

^a^ The data containing 214 antioxidant tripeptides was collected from the literature of Saito et al. [23] and Li et al. [20]. Antioxidant activities of tripeptides were measured by the FTC method and were relative values by adjusting the control to 1.0.

**Table 4:** Sequences and activities of tripeptides on ferric ion reducing antioxidant power (FRAP) dataset ^a^.

No.	Sequence	Activity	No.	Sequence	Activity	No.	Sequence	Activity	No.	Sequence	Activity	No.	Sequence	Activity	No.	Sequence	Activity
1	LTC	2.83	30	LPM	1.04	59	YKK	0.25	88	NGE	−0.30	117	ELK	−0.66	146	KIP	−1.15
2	CQC	2.53	31	TDY	1.01	60	AQA	0.22	89	QSA	−0.33	118	PEQ	−0.66	147	LLD	−1.22
3	GTW	2.52	32	QCH	1.00	61	LRV	0.20	90	DAQ	−0.34	119	IDA	−0.68	148	DLE	−1.22
4	LFC	2.07	33	TWY	0.96	62	PTP	0.18	91	ENS	−0.34	120	LLA	−0.70	149	PEV	−1.22
5	CLV	2.06	34	RVY	0.95	63	ALN	0.18	92	ENG	−0.37	121	ALA	−0.72	150	LKP	−1.40
6	QKW	2.03	35	KWE	0.90	64	LEI	0.16	93	NSA	−0.37	122	GLD	−0.72	151	ALE	−1.52
7	CME	1.99	36	CLL	0.89	65	LVR	0.13	94	EKT	−0.38	123	DIS	−0.72	152	TQL	−1.52
8	YLL	1.91	37	LAM	0.85	66	HIR	0.12	95	EQS	−0.38	124	PEG	−0.72	153	LEE	−1.52
9	QCL	1.69	38	YSL	0.81	67	KKI	0.11	96	AMA	−0.41	125	LDI	−0.74	154	LEK	−1.70
10	LAC	1.69	39	MKG	0.80	68	SFN	0.07	97	KID	−0.41	126	AEP	−0.74	155	DAL	−2.00
11	GEC	1.64	40	QTM	0.80	69	SLL	0.06	98	GAQ	−0.43	127	ALI	−0.77	156	EVD	−2.00
12	EQC	1.52	41	LAL	0.76	70	PAV	0.04	99	PLR	−0.44	128	LDA	−0.77	157	VDD	−2.00
13	FCM	1.51	42	QAL	0.73	71	RLS	0.04	100	ILL	−0.46	129	VFK	−0.77	158	DEA	−2.00
14	CHI	1.45	43	MEN	0.73	72	AGT	0.04	101	VRT	−0.46	130	ALK	−0.77	159	ALT	-
15	ACQ	1.38	44	MKC	0.72	73	LLF	0.02	102	IAE	−0.49	131	AQK	−0.82	160	KGL	-
16	EEL	1.33	45	LSF	0.69	74	PMH	0.00	103	QSL	−0.49	132	IIA	−0.82	161	IQK	-
17	WEN	1.31	46	TCG	0.67	75	EEQ	−0.01	104	KTK	−0.51	133	LIV	−0.85	162	QKV	-
18	VYV	1.19	47	SLA	0.65	76	LVL	−0.02	105	ASD	−0.52	134	EGD	−0.85	163	GDL	-
19	MHI	1.16	48	TMK	0.64	77	QLE	−0.05	106	APL	−0.52	135	QKK	−0.85	164	EIL	-
20	CAQ	1.12	49	LDT	0.62	78	FDK	−0.07	107	AQS	−0.57	136	IPA	−0.85	165	KII	-
21	WYS	1.12	50	EKF	0.54	79	LLL	−0.08	108	ENK	−0.57	137	SDI	−0.89	166	NKV	-
22	KYL	1.08	51	VLV	0.53	80	SAP	−0.08	109	TPE	−0.59	138	VEE	−0.89	167	DTD	-
23	CGA	1.08	52	MAA	0.44	81	LLQ	−0.12	110	RTP	−0.59	139	DDE	−0.89	168	EPE	-
24	KKY	1.08	53	PTQ	0.44	82	NPT	−0.17	111	VLD	−0.62	140	KVL	−0.92	169	EAL	-
25	NEN	1.08	54	VAG	0.41	83	FNP	−0.20	112	IRL	−0.62	141	KFD	−0.92	170	DKA	-
26	ECA	1.07	55	ALP	0.37	84	LNE	−0.24	113	AAS	−0.64	142	IVT	−0.96	171	KAL	-
27	DYK	1.06	56	AVF	0.36	85	SAE	−0.26	114	LQK	−0.64	143	VTQ	−0.96	172	LKA	-
28	KCL	1.05	57	KVA	0.31	86	KPT	−0.28	115	FKI	−0.64	144	AEK	−0.96			
29	YVE	1.05	58	TQT	0.26	87	DIQ	−0.30	116	ISL	−0.66	145	TKI	−1.10	□	□	□

^a^ The data containing 172 antioxidant tripeptides was collected from the literature of Tian et al. [21]. Antioxidant activities of tripeptides were measured by the FRAP assay and were logarithmic transformed. Fourteen inactive peptides were removed before model building.

**Table 5:** Parameters of 16 amino acid descriptors.

Descriptor	No. of Physicochemical Property	No. of Extracted Variable	Scope of Variable
Z-scale [25]	29	3	Electronic property, steric property and hydrophobic property
5Z-scale [26]	26	5	Electronic property, steric property and hydrophobic property
DPPS [27]	119	10	Electronic property, steric property, hydrophobic property and hydrogen bond
MS-WHIM [28]	36	3	Surface charge distribution, size and charge over shape dependence
ISA-ECI [29]	/	2	Isotropic surface area and electronic charge index
VHSE [30]	50	8	Electronic property, steric property and hydrophobic property
FASGAI [31]	335	6	Hydrophobic property, alpha and turn property, bulky property, electronic property, compositional characteristics, local flexibility
VSW [32]	99	9	Molecular size, shape, symmetry and atom distribution
T-scale [33]	67	5	Topological property
ST-scale [34]	827	8	Molecular constitutional, topological, geometrical, hydrophobic, electronic and steric property
E-scale [35]	237	5	Hydrophobic property, size, preferences for amino acids to occur in α-helices, number of degenerate triplet codons and the frequency of occurrence of amino acid residues in β-strands
V-scale [36]	/	3	Van Der Wall’s volume, net charge index and hydrophobic parameter of side chains
G-scale [37]	457	8	Electronic property, steric property and hydrophobic property
HESH [38]	171	12	Electronic property, steric property, hydrophobic property and hydrogen bond
HSEHPCSV [39]	95	12	Hydrophobic, steric, electronic properties and hydrogen bond

## Supplementary Materials

The following are available online at https://www.mdpi.com/1422-0067/20/4/995/s1.

## Abbreviations

BOSS	the bootstrapping soft shrinkage method
DPPH	2,2-diphenyl-1-picrylhydrazyl radical-scavenging capacity
FRAP	ferric-reducing antioxidant power
FTC	ferric thiocyanate
MPA	model population analysis
ORAC	oxygen radical absorbance capacity
PLS	partial least squares
QSAR	quantitative structure-activity relationships
TEAC	Trolox equivalent antioxidant capacity
TRAP	total radical trapping antioxidant parameter
